# The Peripartum Immune Whiplash: Concomitant Cryptococcosis and Granulomatosis With Polyangiitis

**DOI:** 10.7759/cureus.106335

**Published:** 2026-04-02

**Authors:** Thomas Cartwright, Rohit Bhagat, Harjeet Singh, Maria Pino Argumedo

**Affiliations:** 1 Internal Medicine, University of Missouri School of Medicine, Columbia, USA; 2 Pulmonary and Critical Care Medicine, University of Missouri School of Medicine, Columbia, USA

**Keywords:** cryptococcus neoformans (c. neoformans), granulomatosis with polyangiitis (gpa), pr3-anca, pulmonary cryptococcus, pulmonary mass

## Abstract

We present a 19-year-old female, eight weeks postpartum, with a seven-week history of migratory arthralgias followed by progressive dyspnea and pleuritic chest pain. A large mass in her right lower lobe was discovered on computed tomography. Laboratory evaluation demonstrated elevated anti-proteinase 3 (PR3) antibody, along with elevated inflammatory markers. The bronchoalveolar lavage culture grew *Cryptococcus neoformans,* and serum cryptococcal antigen was positive. The patient was diagnosed with pulmonary cryptococcosis and treated with amphotericin and flucytosine, given the severity of her illness. Granulomatosis with polyangiitis was a part of the initial differential but was not diagnosed until she later developed transient hemoptysis, recurrent epistaxis, skin rash, and significant proteinuria. The skin biopsy was consistent with cutaneous vasculitis, and the kidney biopsy was positive for glomerulonephritis, confirming postpartum systemic vasculitis masked by a concurrent pulmonary cryptococcal infection.

## Introduction

Pulmonary nodules and masses in young, otherwise healthy, postpartum women are rare and encompass a broad differential diagnosis, including infection, autoimmune, and malignant etiologies. Granulomatosis with polyangiitis (GPA), formerly known as Wegener’s granulomatosis, is the most common vasculitis in the antineutrophil cytoplasmic antibody (ANCA) associated vasculitides (AAV) spectrum. GPA generally affects small to medium-sized vessels and can be characterized clinically by constitutional symptoms, polyarthralgia and myalgias, sinusitis, epistaxis, hemoptysis, pulmonary nodules, and glomerulonephritis [1]. Diagnosis relies on clinical presentation supported by serologic, radiologic, and histopathologic findings. Infections may confound the diagnosis of AAVs.

Cryptococcus is an invasive fungus transmitted through the inhalation of spores generally found in soil or decaying wood [2]. Presence of a cryptococcal infection is rare in healthy individuals, more frequently occurring in immunocompromised hosts. Pulmonary cryptococcosis typically presents with nonspecific symptoms such as cough, dyspnea, chest pain, fever, and malaise.

During pregnancy, the body shifts its immune state with a decrease in T helper 1 (Th1) cytokines and an increase in T helper 2 (Th2) cytokines, which creates an environment protective to the fetus [3]. It is this same Th2 bias that creates the immunosuppression necessary for a variety of infections, including cryptococcus, to present themselves. This case highlights a rare clinical presentation of concomitant pulmonary cryptococcal infection and new-onset GPA in a postpartum, immunocompetent patient.

## Case presentation

A 19-year-old female, eight weeks postpartum, presented with a two-week history of worsening shortness of breath. She had recently completed antibiotic therapy for presumed pneumonia without improvement. Associated symptoms included pleuritic chest pain, migratory arthralgias, progressive extremity weakness, fever, night sweats, and chills. She denied weight loss. Her medical history was unremarkable, and she denied tobacco use, recreational drug use, or significant environmental exposures.

**Figure 1:**
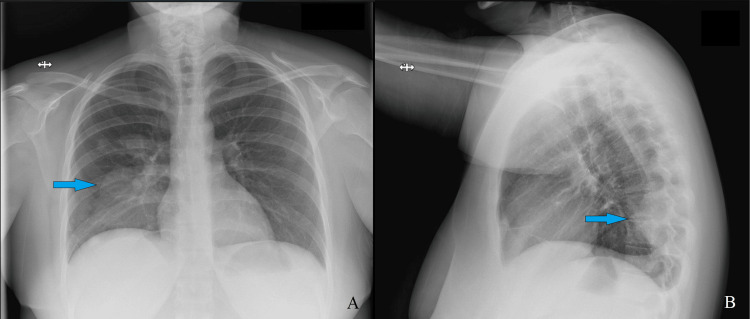
Chest X-ray on admission Posterior-anterior (A) and lateral (B) chest X-ray with arrows showing the right lower lobe opacity.

**Figure 2:**
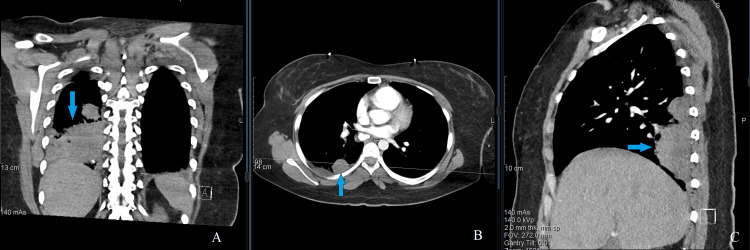
CT chest on admission, in three planes Coronal plane (A), axial plane (B), and sagittal plane (C). Arrows showing lung mass of the right lower lobe measuring 5 × 5 × 3 cm.

On examination, she was tachypneic with crackles at the right lung base. Chest radiography demonstrated a well-circumscribed right lower lobe opacity (Figure 1). Computed tomography (CT) of the chest revealed a 5 × 5 × 3 cm mass-like consolidation in the right lower lobe of the lung (Figure 2). Laboratory studies showed mild leukocytosis, elevated erythrocyte sedimentation rate, and elevated C-reactive protein (Table 1). Respiratory pathogen testing was negative.

**Table 1:** Laboratory results Laboratory results at the time of initial hospital admission, at the time of formal GPA diagnosis, and 30 days after induction of immunosuppressive agents. Ag, antigen; ALT, alanine transaminase; ANCA, antineutrophil cytoplasmic antibody; AST, aspartate transaminase; CRP, C-reactive protein; CSF, cerebrospinal fluid; ESR, erythrocyte sedimentation rate; GPA, granulomatosis with polyangiitis; Hb, hemoglobin; HSV, herpes simplex virus; IgG, immunoglobulin G; IgM, immunoglobulin M; K, potassium; MPO, myeloperoxidase; Na, sodium; Plt, platelet count; PR3, proteinase 3; RBC, red blood cell; WBC, white blood cell

Laboratory Test	Initial Presentation (Day 0)	Diagnosis of Cryptococcal Infection (Day 14)	Diagnosis of GPA (Day 80)	30 Days After Induction of Rituximab and Methylprednisolone (Day 110)	Reference Range
WBC (× 10⁹/L)	10.69	16.36	7.20	7.18	3.50-10.50
Hb (g/dL)	11.4	10.1	8.4	10	12-15
Plt (× 10⁹/L)	527	420	298	354	150-450
Na (mmol/L)	140	139	137	140	136-145
K (mmol/L)	3.0	3.4	3.4	3.8	3.5-5.1
Creatinine (mg/dL)	0.8	0.7	0.9	0.92	0.5-1.0
AST (U/L)	30	16	38	13	≤34
ALT (U/L)	21	30	30	<7	10-40
ESR (mm/hour)	117	13	> 130	17	0-20
CRP (mg/dL)	15.6	<0.5	28.1	0.5	≤0.5
ANCA Titer	1:1280	-	1:1280	Not tested	-
Anti-MPO (CU)	4.1	-	< 3.2	Not tested	≤20
Anti-PR3 (CU)	1,002.2	-	1596.1	612.9	≤20
Cryptococcal Ag, Serum	-	Positive	Negative	-	-
HIV 1,2 Antigen Antibody, Serum	-	Non-reactive	Non-reactive	-	-
Syphilis Antibody IgG and IgM, Serum	-	Non-reactive	-	-	-
QuantiFERON Gold, Serum	-	Negative	-	-	-
CSF, Cryptococcal Ag	-	Negative	-	-	-
CSF, HSV Type 1/2 DNA	-	Not Detected	-	-	-
CSF, Protein (mg/dL)	-	34	-	-	15-45
CSF, Albumin (mg/dL)	-	9.9	-	-	≤27
CSF, Glucose	-	54	-	-	40-70
CSF, WBC (/mcL)	-	0	-	-	0-30
CSF, RBC (/mcL)	-	0	-	-	-
CSF, IgG (mg/dL)	-	1.2	-	-	≤8.1

Pulmonology performed a flexible bronchoscopy with transbronchial biopsy of the right lower lobe mass. Bronchoalveolar lavage (BAL) samples were obtained from the right middle and lower lobes prior to biopsy. Autoimmune evaluation revealed a positive ANCA titer with markedly elevated anti-PR3 levels. Serologic testing for *Histoplasma*, *Blastomyces*, and *Bartonella* species was negative. Given the significantly elevated anti-PR3 levels and concern for GPA, immunosuppressive therapy was considered; however, definitive therapy was deferred until results of cultures and fungal testing returned. Given the patient's severe joint pain, which limited daily activity, a seven-day course of high-dose prednisone was initiated as a temporizing measure, and the patient was discharged.

Results of the transbronchial biopsy showed a mixed inflammatory infiltrate with multinucleated giant cells and neutrophils. There was no evidence of hemorrhage or necrotizing vasculitis, suggesting an infectious process; however, stains for microorganisms were negative. BAL cultures subsequently grew *Cryptococcus neoformans*, prompting hospital readmission. At that time, she reported new-onset photophobia, blurry vision, and neck stiffness, concerning for meningitis. Serum cryptococcal antigen was positive, although cerebrospinal fluid studies and cultures were negative. Prednisone was discontinued, and induction therapy with liposomal amphotericin B and flucytosine was initiated for a total of seven days. The patient was later transitioned to oral fluconazole upon discharge with an intended duration of six months.

**Figure 3:**
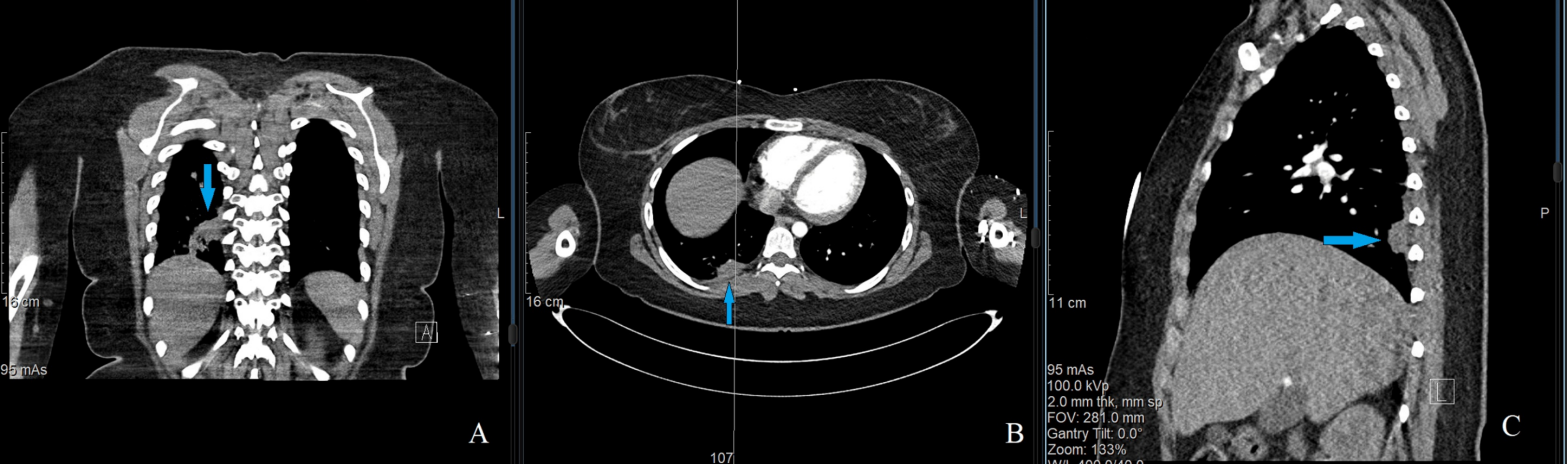
Chest CT after anti-fungal therapy, in three planes Coronal plane (A), axial plane (B), and sagittal plane (C). Arrows showing lung mass of the right lower lobe after antifungal treatment, now measuring 2.9 × 2 × 1.5 cm.

**Figure 4:**
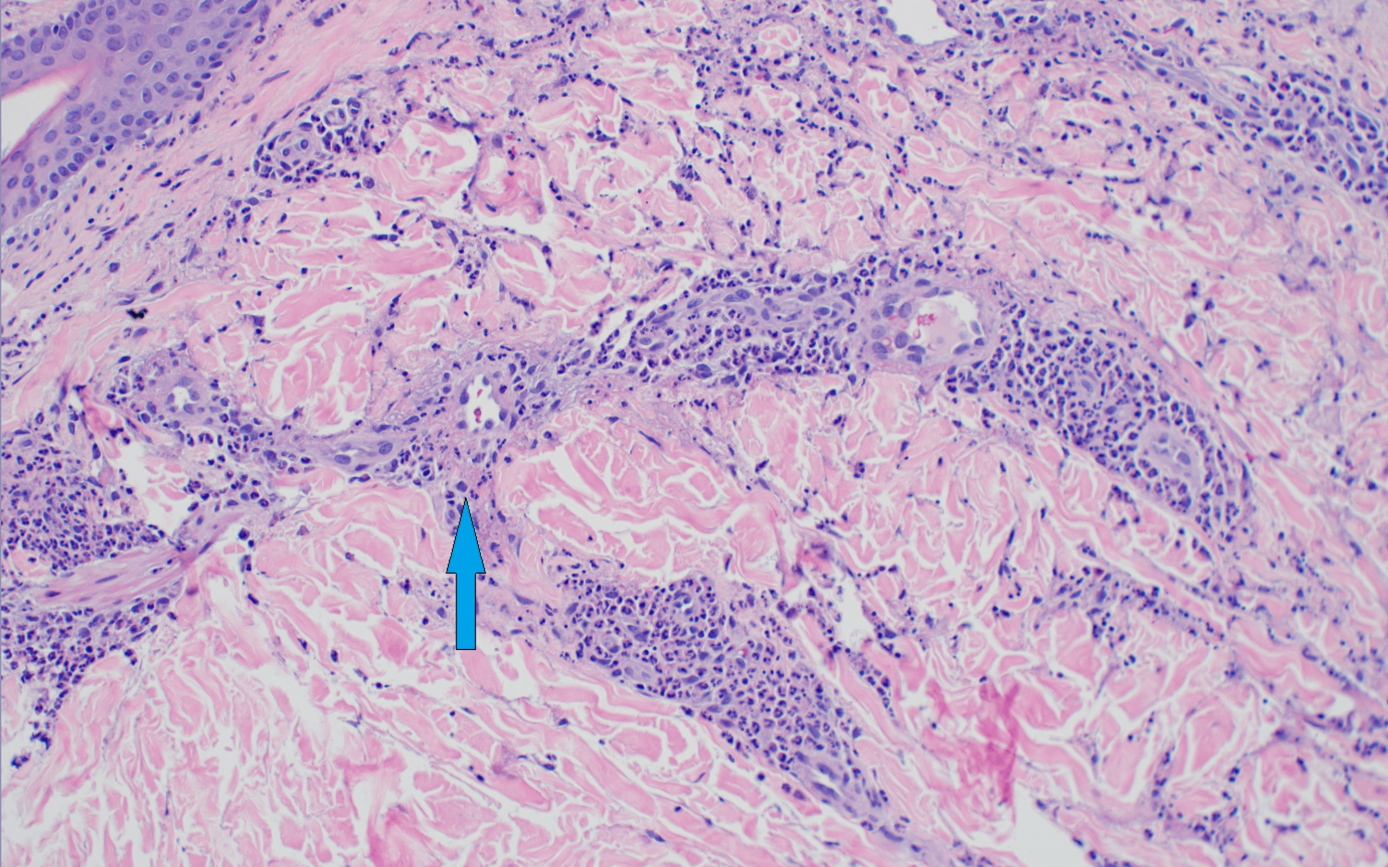
Skin biopsy from the upper arm Arrow demonstrating cutaneous small vessel vasculitis, which is signified by neutrophils in the vessel wall and fibrinoid necrosis of the vessel wall.

**Figure 5:**
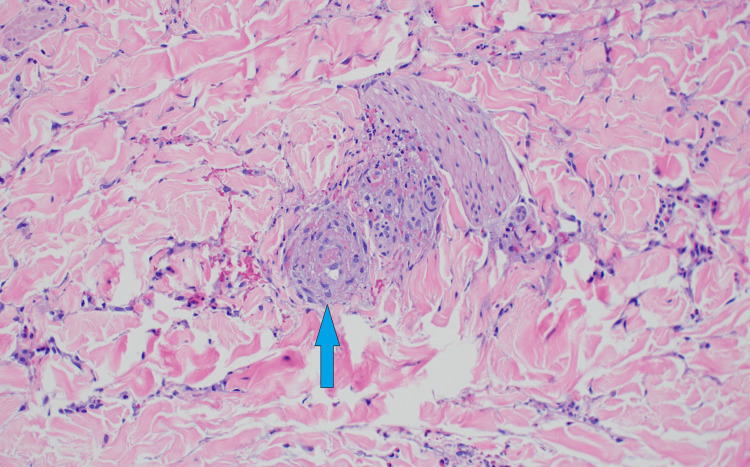
Skin biopsy from the lower leg Arrow demonstrating cutaneous small vessel vasculitis, which is signified by neutrophils in the vessel wall and fibrinoid necrosis of the vessel wall.

Two months later, she returned with a diffuse rash involving all four extremities. She also reported worsening myalgias, recurrent epistaxis, oral lesions, syncope, conjunctival injection, and myodesopsia. Repeat CT imaging of the chest demonstrated interval reduction of the prior pulmonary mass to 2.9 × 2 × 1.5 cm (Figure 3). Skin biopsy of the rash, taken from upper and lower extremities, demonstrated fibrinoid necrosis of small vessels within the superficial and deep dermis, with neutrophilic infiltration consistent with small-vessel vasculitis (Figures 4-5). Serum cryptococcal antigen was now negative while anti-PR3 remained positive. Twenty-four-hour urine collection revealed proteinuria exceeding 1 g. Kidney biopsy demonstrated focal crescentic glomerulonephritis consistent with PR3-ANCA-associated vasculitis, confirming the diagnosis of GPA. She was subsequently treated with rituximab and intravenous methylprednisolone, as well as started on trimethoprim/sulfamethoxazole for Pneumocystis jirovecii pneumonia prophylaxis. She continued on an outpatient infusion regimen for an additional two treatments; however, she became pregnant, and treatment, as well as further imaging, was placed on hold until after delivery. The patient has since been lost to follow-up. 

## Discussion

Cryptococcal infection typically affects immunocompromised hosts; however, pregnancy and the early postpartum period represent transient states of immune modulation that may predispose otherwise healthy women to opportunistic pathogens. During pregnancy, elevated progesterone, estradiol, and cortisol shift the maternal immune system toward a tolerogenic, Th2-dominant profile, suppressing Th1 cytokines such as IL-12 and TNF-α while enhancing regulatory T-cell activity [3,4]. Although protective for the semi-allogenic fetus, this environment impairs cell-mediated immunity and macrophage function, increasing susceptibility to fungal infections. Following delivery, rapid hormonal decline leads to immune reconstitution characterized by restoration of Th1 activity, increased proinflammatory cytokine production, and an elevated Th17/Treg ratio [3,5,6]. This shift toward a pro-inflammatory, autoreactive immune environment is recognized as a trigger for new-onset or relapsing autoimmune disease, as all forms of primary systemic vasculitis have been reported in the postpartum period, GPA being one of the more common [7-9]. This immunologic framework provides a unifying explanation for our patient’s dual presentation: impaired pathogen clearance in late pregnancy likely permitted pulmonary cryptococcosis to develop, while the subsequent postpartum immune rebound unmasked underlying PR3-ANCA-mediated autoimmunity.

Additionally, interpretation of the markedly elevated PR3 levels is challenging, as prolonged infections can mimic AAV. However, such false positives are more commonly associated with bacterial and viral infections than fungal organisms [10]. When infections precede AAVs, myeloperoxidase (MPO)-associated disease is more frequently observed than PR3-associated disease [11]. Prior reports have described elevated PR3 in biopsy-proven pulmonary cryptococcosis without evidence of systemic vasculitis, but these accounts are rare [12,13]. Further complicating interpretation is inter-assay variability in PR3 testing, as no standardized cutoff ensures optimal specificity. Studies suggest that titers of three to ten times the upper limit of normal yield specificities between 78% and 90%, while a 2021 meta-analysis reported a pooled specificity of 98% for ANCA testing in confirmed AAV, though without defined cutoff thresholds [14-16]. In contrast to isolated serologic elevation, our patient developed biopsy-confirmed small-vessel vasculitis and crescentic glomerulonephritis, supporting a true diagnosis of PR3-positive GPA following control of infection.

## Conclusions

This case illustrates that in the postpartum period, immune dysregulation can give rise to two seemingly distinct pathologies: opportunistic fungal infection and ANCA-associated vasculitis. Comprehensive imaging and invasive testing enabled the timely identification of pulmonary cryptococcosis, yet a concurrent, evolving vasculitis remained masked until the infection was treated and immune function continued to rebound. Clinicians should recognize that the peripartum immune shift can simultaneously impair pathogen clearance and unmask autoimmune processes, allowing conditions such as cryptococcosis and GPA to coexist. Opportunistic infections should be considered in young postpartum patients with elevated autoimmune markers, while avoiding premature exclusion of autoimmune disease.
